# Editorial: Digital solutions to HPV vaccination

**DOI:** 10.3389/fdgth.2022.972234

**Published:** 2022-08-09

**Authors:** Suellen Hopfer, Amalie Dyda, Heather M. Brandt

**Affiliations:** ^1^ Department of Health, Society & Behavior, Program in Public Health, University of California, Irvine, CA, USA; ^2^ School of Public Health, University of Queensland, Herston, Australia; ^3^ Director, HPV Vaccine Cancer Prevention Program, St. Jude Comprehensive Cancer Center, Memphis, TN, United States

**Keywords:** HPV, vaccination, social media, youth, parents, equity, digital

Editorial on the Research Topic Digital solutions to HPV vaccination by Hopfer S, Dyda A and Brandt HM. (2022). Front. Digit. Health. 4:972234. doi: 10.3389/fdgth.2022.972234

## Introduction

The rapid growth and diffusion of digital solutions, technologies and online media consumption is changing the landscape of implementing health behavior change campaigns and interventions as well as how individuals and families access and consume health information (1, 2). Novel, culturally targeted prevention strategies can be implemented digitally to reach virtual communities who access digital health messages daily especially young adults and youth (3–7). Technology may also improve health care more directly, with text-message reminders and other recall systems showing improvements in health outcomes. Digital solutions hold promise for raising awareness about health promotion, empowering individuals and delivering real-time health information that can be tailored to individual needs. Prioritizing digital health for vaccine information has been reflected in US federal public health goals for 2030 including health equity goals (8). We focus in this special issue on digital solutions to increasing HPV vaccination, a priority area for cancer prevention (9). The US federal National Cancer Institute (NCI) designated cancer centers have called for urgent back on track vaccine initiatives to attenuate the gap in HPV vaccination (10) that show 2.3 million doses of HPV vaccine missed (a decrease of greater than 20%) during the COVID-19 pandemic and is likely to persist for several years post-pandemic (11).

This special issue showcases predominantly social media studies as digital solutions that show promise for reaching parents and youth about HPV vaccination (Massey et al.; Zhang et al.; Sundstrom et al.; Buller et al.; Hopfer et al.). The special issue also presents feasibility studies on web-based or mobile applications (Woodall et al.; Olusanya et al. and Reno & Dempsey) that tailor vaccine information to parents, adolescents, and young adults through a variety of approaches. Most digital solutions presented are stand-alone, yet one intervention presents digital efforts integrated as a component of a multi-level clinic intervention to reach rural populations and reduce missed HPV vaccine recommendation opportunities (Kepka et al.). Other studies (Woodall et al.; Olusanya et al.) suggest that mobile apps could be used to complement clinical pediatric well-child visits or could be disseminated by state health departments, school health officials, and pharmacy chains. The advantages of digital health not only become apparent by the structural channel affordances that lend themselves to greater engagement and tailoring with audiences but also accommodate busy schedules of parents and young adults.

Digital approaches inherently hold promise to make vaccine information more accessible for many populations who might otherwise not be reached and where messaging can be targeted to reach subgroups. Digital strategies may be delivered *via* peer networks (e.g., mothers of vaccinated adolescents as champions or influencers), such as those reported by Sundstrom et al. and Buller et al., to deliver vaccine messaging through trusted peers presents a promising approach for increasing HPV vaccination. Another benefit of digital interventions is the ability to deliver vaccine messaging in other languages e.g., Spanish to Latinx communities (Reno & Dempsey, CHiCOS; Woodall et al. Vacteens/Vacunadolescente.org) or target vaccine messaging *via* parent personas (Massey et al.). Social media studies delivered vaccine messaging across multiple channels testing the impact of disseminating through different platforms (Hopfer et al.) in private and public groups, synchronous/asynchronous discussion groups, webinars, bi-weekly emails, weekly messaging, and lifestyle messaging (Massey et al., Olusanya et al.; Sundstrom et al.; Hopfer et al.).

Geographic targeting can also be achieved with digital messaging: one study focused on Northern rural California with low vaccination and high cancer rates (Zhang et al.), another study focused regionally on South Carolina (Sundstrom et al.) and yet another on rural Colorado (eno & Dempsey). These U.S. regions are characterized by low HPV vaccination rates and high HPV-cancer incidence.

Engagement, as observed through analysis of comments and posts (Zhang et al.; Buller et al.), showcases the potential to take advantage of answering parent and youth questions real-time and debunking misinformation, but also answering questions asynchronously through newsletters, blogs, discussion forums, chat rooms, and portals (Sundstrom et al.; Hopfer et al.). A devoted moderator who can respond real-time to questions and correct misinformation was a recurring theme in combination with more automated digital interventions. The flow of unsolicited, real-time comments from parents give insight into vaccine concerns as well as exposure to circulating rumors and misinformation. Predominant vaccine concerns remain around HPV vaccine confidence and HPV complacency.

Communication and behavior change theories were adapted to understand the digital health environment: these ranged from social cognitive theory (SCT), diffusion of innovation (DOI), extended parallel process model (EPPM), 3C model of vaccine hesitancy, and a champion network approach. One study integrated persona theory with health communication and human-centered design (Massey et al.) while another study applied dialog theory to segment HPV vaccine videos and adapt them to the social media environment (Hopfer et al.). An ideal area of opportunity lies in strengthening the integration of theoretical foundations into digital health solutions in HPV vaccination.

A number of these studies were in the development or piloting stage (Olusanya et al.), highlighting the emerging nature of digital solutions to increase vaccination. However, data on outcomes was also presented with results from randomized trials (Woodall et a.l, Buller et al.). This accrued evidence from the studies in this special issue on digital solutions highlights the various ways in which digital approaches are likely to increase HPV vaccination rates particularly in relation to increasing access and improving health promotion reach, through peer- and trusted channels and messengers. Use of narratives and informal peer dialogue (Massey et al.; Hopfer et al.; Sundstrom et al.) show promise to increase user engagement in the digital environment while also being able to answer and debunk circulating rumors real-time (Sundstrom et al.). Social media platforms offer one strategy to make information accessible (Zhang et al.). Additional strategies of using peer-champions (Sundstrom et al.), point-of-care delivered vaccine messages (Kepka et al.), and real-time tailoring (Hopfer et al.) also show promise as digital solutions to increase HPV vaccination.
